# Circular RNA TAF4B Promotes Bladder Cancer Progression by Sponging miR-1298-5p and Regulating TGFA Expression

**DOI:** 10.3389/fonc.2021.643362

**Published:** 2021-07-12

**Authors:** Xiaoting Zhang, Xiaofeng Li, Xian Fu, Mengli Yu, Guicheng Qin, Guihong Chen, Chenchen Huang

**Affiliations:** ^1^ Shenzhen Bao’an District Songgang People’s Hospital, Shenzhen, China; ^2^ Department of Urology, Peking University First Hospital, Beijing, China; ^3^ Department of Urology, Peking University Shenzhen Hospital, Shenzhen, China; ^4^ Department of Laboratory Medicine, Peking University Shenzhen Hospital, Shenzhen, China; ^5^ School of Pharmaceutical Sciences, Guangzhou Medical University, Guangzhou, China

**Keywords:** bladder cancer, circTAF4B, miR-1298-5p, TGFA, progression

## Abstract

**Background:**

Bladder cancer (Bca) is the most common malignant tumor of the urinary system. Circular RNAs (circRNAs) have been recognized as key regulators in tumorigenesis. However, the molecular mechanisms underlying circRNAs involved in the progression of BCa remain largely unknown.

**Methods:**

We detected the expression level of circular RNA TAF4B (circTAF4B) by qRT-PCR assay. Cell proliferation was evaluated by CCK-8 and colony formation assays. Wound healing and Transwell assays were performed to measure cell migration and invasion capability. Moreover, we performed qRT-PCR and western blotting assays to determine the expression levels of epithelial-mesenchymal transition (EMT) markers. A nuclear/cytoplasmic fractionation assay was used to measure the subcellular location of circTAF4B. RNA pull-down and dual-luciferase reporter assays were used to detect the target microRNA of circTAF4B. A mouse xenograft model was produced to analyze the effect of circTAF4B on the tumorigenesis of BCa.

**Results:**

In this study, we identified a novel circular RNA, circTAF4B, that is significantly upregulated in BCa and correlated with poor prognosis. Downregulated circTAF4B abolished the growth, metastasis and EMT process in BCa cells. Mechanistically, we found that circTAF4B facilitated the expression of transforming growth factor A (TGFA) by sponging miR-1298-5p. Finally, circTAF4B enhanced the proliferation and EMT process of BCa cells *in vivo*.

**Conclusion:**

In summary, our study demonstrated that circTAF4B played a carcinogenic role in the growth, metastasis, and EMT process of BCa by regulating the miR-1298-5p/TGFA axis. Thus, circTAF4B may become a diagnostic and therapeutic target for BCa.

## Introduction

Bladder cancer (Bca) is the ninth most common cancer in the world and one of the most common malignancies of the urinary system (1, 2). A total of 74000 patients were diagnosed with BCa in the USA, and more than 430000 patients were diagnosed with this disease worldwide in 2017 (3). BCa is divided into two types based on the depth of tumor invasion: nonmuscle-invasive bladder cancer (NMIBC) and muscle-invasive bladder cancer (MIBC) (4–6). Although MIBC accounts for approximately 25% of this disease, nearly half of patients develop tumor metastasis and have a poor prognosis (7, 8). The 5-year survival rate of BCa patients remains at a low level despite the development of surgical and chemotherapy treatments in the past decade (9, 10). However, the mechanisms underlying BCa development and metastasis remain unknown. Therefore, it is urgent to discover novel biomarkers and therapeutic targets for BCa.

Circular RNAs (circRNAs) are a class of noncoding RNAs formed by reverse splicing of premRNA transcript products, which are stably and abundantly maintained in cells, tissues and organs (11, 12). A circRNA is a covalently enclosed circular sequence without a 5’ cap or 3’ polyadenylated tail (13). Due to the development of high-throughput sequencing and circRNA investigation techniques, a wealth of functional circRNAs have been discovered (13, 14). Previous studies showed that circRNAs can interact with microRNAs (miRNAs) to regulate the expression of downstream target genes (15, 16). For instance, circPSMC3 suppresses the proliferation and metastasis of gastric cancer by acting as a ceRNA to sponge miR-296-5p (17). In our previous study, we found that circTLK1 promotes the progression of renal cell carcinoma by sponging miR-136-5p (18). CircRNA MYLK acts as a ceRNA to promote bladder cancer progression (19). However, the role of circRNAs in tumorigenesis and metastasis remains unclear.

It is well known that circRNAs bind to miRNAs with miRNA response elements (MREs) during the growth and metastasis of various cancers. MiRNAs have been demonstrated to play a crucial role in the growth and metastasis of various tumors (20, 21). For instance, miRNA-1298-5p can inhibit the development and progression of BCa and gastric cancer (GC) (22, 23). miR-1298-5p was downregulated in nonsmall cell lung cancer (NSCLC) and correlated with poor prognosis (24). In breast cancer, miR-1298-5p inhibited cell growth and metastasis by suppressing CXCL11 expression.

In this study, we focused on circ_0047322 by screening RNA-Sequence data from a previous study (25). circ_0047322 is derived from the TATA-box binding protein (TBP)-associated factor 4B (TAF4B) gene and was identified as circTAF4B. circTAF4B was significantly upregulated in BCa tissues and closely correlated with poor prognosis in BCa patients. More importantly, we found that circTAF4B sponges miR-1298-5p in a ceRNA-dependent manner to modulate transforming growth factor A (TGFA) expression, thus promoting the proliferation, migration, invasion and EMT process of BCa. Our findings revealed a novel potential mechanism by which the circTAF4B/miR-1298-5p/TGFA signaling pathway is involved in the tumorigenesis and metastasis of BCa.

## Materials and Methods

### Patients and Clinical Samples

A total of 76 pairs of tumor tissues and matched adjacent normal tissues were collected from BCa patients who underwent surgery. The clinical data of 76 bladder cancer patients are shown in (**Table 1**, **Supplementary Table S3**). This study was approved by the Regional Committee for Medical Research Ethics and the Human Ethics Committee of Peking University Shenzhen Hospital. All Bca patients approved this study.

**Table 1:** Correlation between circTAF4B expression and clinical characteristics in 76 BCa patients.

Characteristics	Total	circTAF4B expression		P value
		High	Low	
Gender				
Male	48	34	14	0.509
Female	28	16	12	
Age (years)				
<60	36	21	15	0.231
≥60	40	29	11	
Tumor size (cm)				
<3cm	31	18	13	0.326
≥3cm	45	32	13	
Histological grade				
Low	27	12	15	0.005**
High	49	38	11	
T stage				
T_1_/T_2_	44	25	19	0.086
T_3_/T_4_	32	25	7	
Lymphatic metastasis				
Yes	12	11	1	0.049*
No	64	39	25	
Postoperative metastasis				
Yes	19	13	5	0.581
No	57	37	21	

*P < 0.05 and **P < 0.01 represent statistical significance.

Results are analyzed by Chi-square test.

### Cell Lines

All cell lines, including SV-HUC1 and BCa cell lines, were obtained from the American Type Culture Collection (ATCC, Manassas, VA, USA). BCa cell lines were cultured in DMEM (Invitrogen, Carlsbad, CA, USA) containing 10% fetal bovine serum (FBS) and 1% antibiotics. SV-HUC1 cells were grown in F12K medium mixed with 10% FBS and 1% antibiotics. All cell lines were cultured in an incubator at a temperature of 37°C with 5% CO_2_.

### RNA Extraction and Quantitative Real-Time PCR

TRIzol reagent (Invitrogen, Carlsbad, CA, USA) was used to extract total RNA. Quantitative real-time PCR (qRT-PCR) was performed according to a SYBR Green PCR kit (Takara) and conducted on a Roche LightCycler^®^ 480II PCR instrument (Basel, Switzerland). GAPDH or U6 nuclear RNA was applied as an internal control. The sequences of the primers used in this research are listed in **Supplementary Table S1**.

### Transfection

Two short hairpin RNAs (shRNAs) targeting circTAF4B (shcirc-1, shcirc-2) and the corresponding control (shCtrl) were devised by GenePharma and cloned into the pGPU6/GFP/Neo vector. The sequences of oligonucleotides are listed in **Supplementary Table S3**. miR-1298-5p mimic and its corresponding control (miR-NC) and miR-1298-5p inhibitor and its corresponding control (NC inhibitor) were purchased from RiboBio. The overexpression vector for TGFA (pcDNA3.1-TGFA) and the corresponding control (pcDNA3.1-NC) were synthesized by GenePharma. Lipofectamine 3000 reagent (Invitrogen) was used for all transfections according to the instructions. For stable transfection, SW780 cells were infected with lentivirus with shcirc-1, which was previously verified to be effective, or its corresponding control (shCtrl) and then selected with 3 μg/mL puromycin for two weeks.

### Cell Proliferation Assay

Cell Counting Kit-8 (CCK-8) (Beyotime Institute of Biotechnology) experiments and colony formation experiments were used to evaluate cell proliferation ability. For the CCK-8 experiment, the transfected BCa cells were cultured in a 96-well plate at a concentration of 1000 cells per well. The absorbance value of BCa cells was measured at 450 nm using a microplate analyzer (Bio-Rad, Hercules, CA, USA) at 0, 24, 48, 72, and 96 hours. For the colony formation assay, BCa cells were cultured in 6-well plates at a concentration of 1000 cells per well for 15 days. The colonies were stained with crystal violet and rinsed with glacial acetic acid. Finally, the absorbance value was measured at 595 nm by using a microplate reader.

### Wound Healing Assay

A wound healing assay was used to measure cell migration ability. The transfected Bca cells were grown in 6-well plates. A yellow pipette tip was used to draw scars until cells grew to 90-95% abundance. After 24 hours, we used a digital microscope at 10-fold magnification to capture images of the migrated cells.

### Transwell Assay

Transwell assays were used to evaluate the migration and invasion ability of BCa cells. Transfected cells were mixed in serum-free medium and inoculated in Transwell inserts (Corning) coated with matrix gel (BD Biosciences) or left uncoated at a density of 2-3×10^4^ cells/well. Medium containing 10% serum was added to the lower chamber. After incubation for 24-48 hours, cells were stained with 0.1% crystal violet and washed with PBS. Observation and photography were conducted under a microscope at 20x.

### Western Blotting Assay

Protein was extracted with RIPA reagent (Beyotime, Beijing, China), separated by 10% SDS-PAGE, and transferred to PVDF membranes. The PVDF membranes were blocked with 5% milk for one hour and incubated with primary antibody at 4°C for over 12 hours and secondary antibody at room temperature for 1 hour. Quantity One software (Bio-Rad) was used for luminescent detection and imaging. N-cadherin, vimentin, E-cadherin, and GAPDH antibodies were provided by Cell Signaling Technology (Danvers, MA, USA), and TGFα antibody was obtained from Abcam (USA).

### Nuclear/Cytoplasmic Fractionation

Nuclear/cytoplasmic fractionation was performed using a PARIS Kit (Life Technologies, MA). After the nuclei and cytoplasm were separated according to the instructions, the expression of the target gene was detected by qRT-PCR, and GAPDH and U6 were used as controls.

### RNA Pull-Down Assay

SW780 and T24 cells were cotransfected with pcDNA3.1-circTAF4B and biotin-labeled circTAF4B. The pull-down proteins were treated with RNase-free DNase I and an RNeasy Mini Kit (QIAGEN, Germany). microRNAs were extracted and quantitatively detected by qRT-PCR. The primers applied in this experiment and their corresponding sequences are listed in **Supplementary Table S1**.

### Dual-Luciferase Reporter Assay

A luciferase reporter plasmid was designed and synthesized by GeneCopoeia. The sequences of wild-type (WT) circTAF4B or mutated (Mut) circTAF4B were cloned into the MT06 vector. The sequences of wild-type (WT) TGFA and mutated (Mut) TGFA were cloned into the MT07 vector. Luciferase reporter plasmids and miR-1298-5p were cotransfected into BCa cells. Finally, the luciferase activity of BCa cells was measured by using a Luciferase Reporter Kit (Promega).

### Tumor Xenografts

Four-week-old male BALB/C nude mice were used for the tumor xenotransplantation assay in this study. Twelve mice were randomly divided into two groups. SW780 cells expressing shcircTAF4B (5×107) were injected subcutaneously into the backs of mice. The volume of tumor xenografts was measured weekly. Six weeks later, the mice were sacrificed, and the tumors were collected and weighed. Total RNA and total protein were extracted from tumors to detect the expression of target genes.

### Statistical Analysis

All data from independent replications were analyzed using SPSS 22.0 (SPSS), and the results are expressed as the mean ± standard deviation (SD). The difference between groups was analyzed by Student’s t test. A P value < 0.05 or a P value <0.01 was considered statistically significant.

## Results

### circTAF4B Is Significantly Augmented in BCa Tissues and Positively Associated With Poor Prognosis

**Figure 1:**
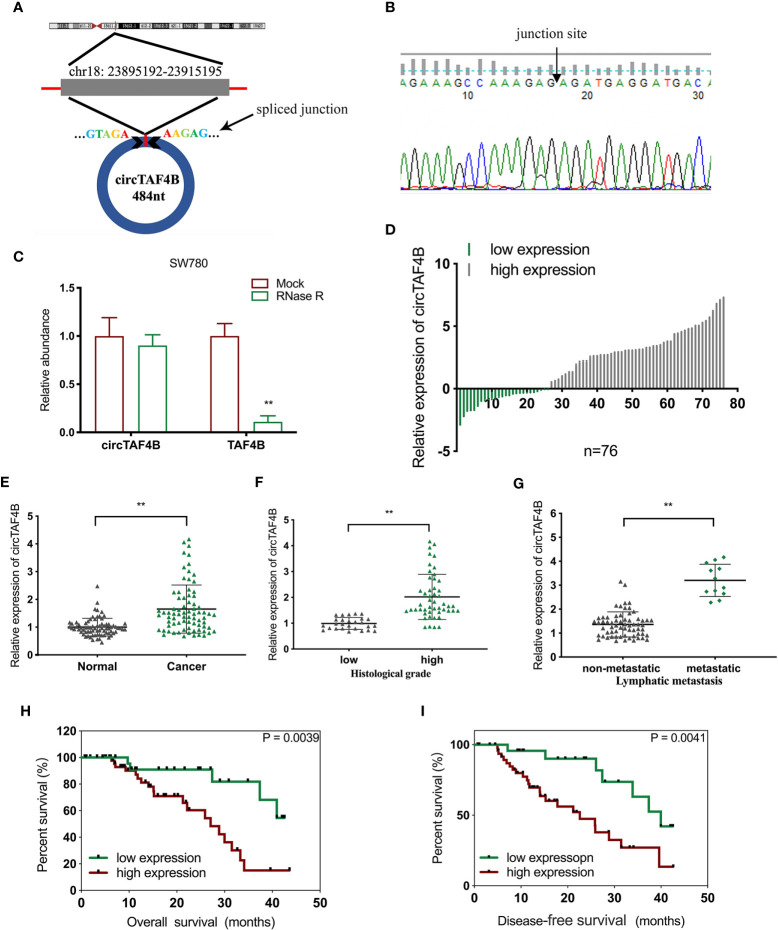
circTAF4B was overexpressed in BCa tissues and associated with clinicopathological characteristics. **(A)** Schematic diagram of the circTAF4B structure and spliced junction. **(B)** The junction site of circTAF4B was confirmed by Sanger sequencing. **(C)** Compared with TAF4B mRNA, circTAF4B was not degraded by RNase R enzyme. **(D, E)** The expression levels of circTAF4B in tumor and adjacent normal tissues. **(F)** Differences in circTAF4B expression between tissues with high and low pathological grades of bladder cancer. **(G)** Differences in circTAF4B expression between patients with and without lymphatic metastasis. **(H)** Differences in overall survival time (OS time) between the circTAF4B high expression group and the circTAF4B low expression group. **(I)** Differences in disease-free survival (DFS) between the circTAF4B high expression group and the circTAF4B low expression group. Data represent the mean ± SD of more than three independent experiments. *p < 0.05; **p < 0.01.

Through a bioinformatics website (circBank), we determined that circTAF4B (circ_0047322) is 484 nt in length and formed by the reverse splicing of TAF4B mRNA, which is located at CHR18: 23895192-23915195. The splicing site of circTAF4B is shown in **Figure 1A**. Sanger sequencing was applied to verify the junction site (**Figure 1B**). The RNase R enzyme digestion assay demonstrated that circTAF4B was resistant to RNase R instead of TAF4B mRNA (**Figure 1C**). Next, we discovered that circTAF4B was significantly augmented in 65.7% (50/76) of BCa tissues (**Figure 1D**). The expression level of circTAF4B in tumor tissues was also significantly increased compared to that in matched adjacent normal tissues (**Figure 1E**). In addition, increased expression of circTAF4B was positively correlated with higher histological grade and lymphatic metastasis (**Table 1**, **Figures 1F, G**). However, high expression of circTAF4B was associated with a lower overall survival rate and disease-free survival rate of bladder cancer patients (**Figures 1H, I**), suggesting that circTAF4B may be a cause of the poor prognosis of BCa patients.

### Knockdown of circTAF4B Inhibits Proliferation, Metastasis, and EMT of Bca Cells *In Vitro*

**Figure 2:**
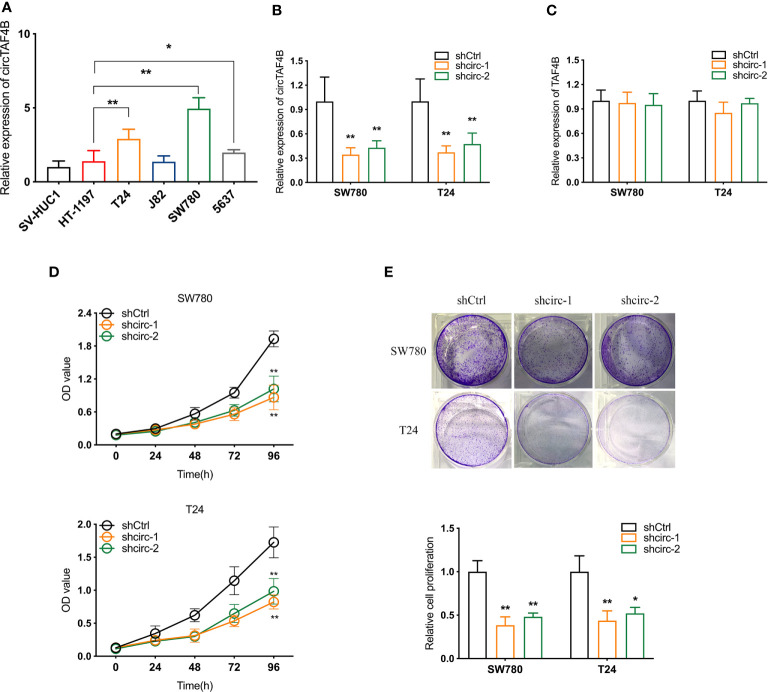
Knockdown of circTAF4B inhibited the proliferation of BCa cells *in vitro*. **(A)** The expression level of circTAF4B in different Bca cell lines and a normal epithelial cell line. **(B, C)** The effect of shRNAs targeting circTAF4B (shcirc-1 and shcirc-2) on circTAF4B and TAF4B expression. **(D, E)** CCK-8 assay and colony-formation assay showed that transfection of circTAF4B decreased the proliferation of BCa cells. Data represent the mean ± SD of more than three independent experiments. *p < 0.05; **p < 0.01.

**Figure 3:**
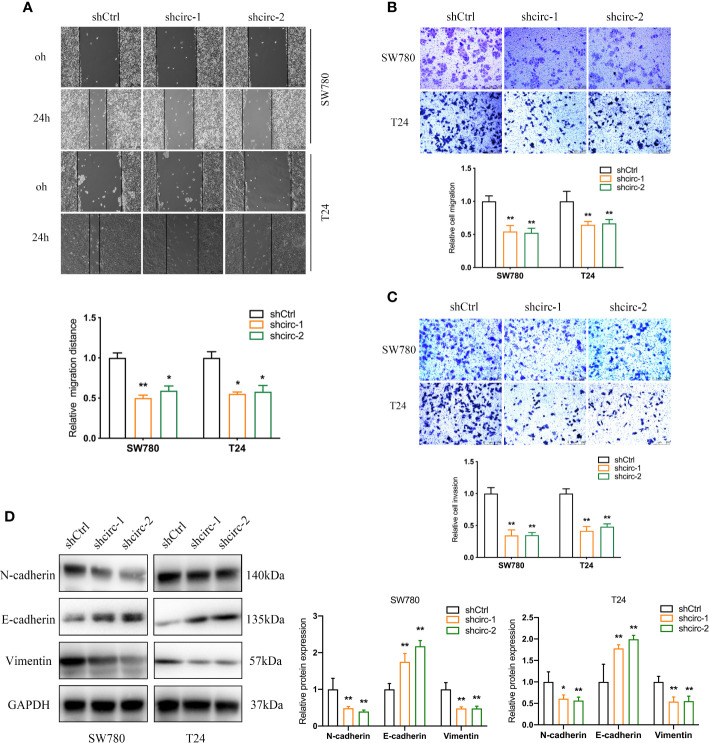
Knockdown of circTAF4B suppressed the migration, invasion and EMT process of BCa cells *in vitro*. **(A, B)** Wound healing and Transwell migration tests showed that transfection of shcircTAF4B downregulated the migration of BCa cells. **(C)** Transwell invasion assays revealed that knockdown of circTAF4B inhibited the invasion of BCa cells. **(D)** Protein expression levels of EMT markers were detected by western blotting assay. Data represent the mean ± SD of more than three independent experiments. *p < 0.05; **p < 0.01.

Compared to the urinary bladder cell line HT1197, the circTAF4B expression level was obviously increased in BCa cells, especially SW780 and T24 cells (**Figure 2A**). Hence, SW780 and T24 cells were selected for further experiments. To explore the role of circTAF4B in the development of BCa, we synthesized an shRNA targeting circTAF4B to suppress circTAF4B expression (**Figure 2B**). However, knockdown of circTAF4B could not modulate TAF4B mRNA expression, suggesting that circTAF4B could not regulate the transcription of TAF4B (**Figure 2C**). CCK-8 and colony formation assays showed that knockdown of circTAF4B significantly inhibited the proliferation of BCa cells (**Figures 2D, E**). Wound healing and Transwell migration assays demonstrated that inhibition of circTAF4B significantly reduced the migration ability of BCa cells (**Figures 3A, B**, **6B**). Transwell invasion assays showed that downregulation of circTAF4B could reduce the invasion ability of BCa cells (**Figure 3C**). Furthermore, knockdown of circTAF4B significantly inhibited the expression of N-cadherin, Snail and Vimentin and increased E-cadherin expression (**Figure 3D**), indicating that circTAF4B promoted the EMT process.

### circTAF4B Predominantly Sponges miRNA-1298-5p

**Figure 4:**
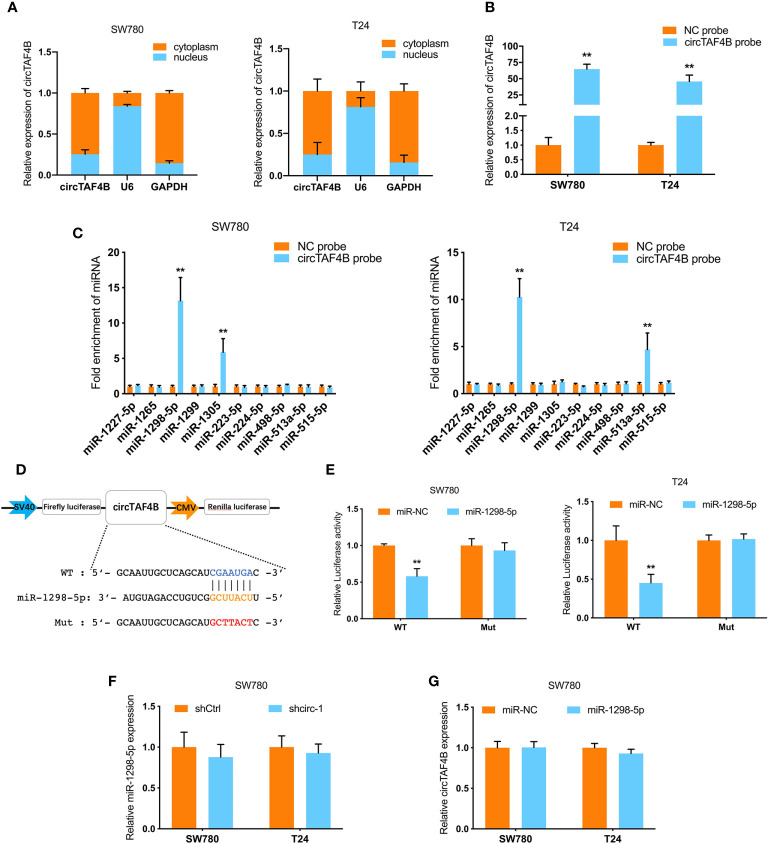
circTAF4B sponges miR-1298-5p. **(A)** The distribution of circTAF4B in the cytoplasm and nucleus was detected by nuclear/cytoplasmic fractionation experiments. **(B)** The efficiency of the biotin-labeled circTAF4B probe was verified by qRT-PCR. **(C)** RNA pull-down assay showed that the enrichment of microRNAs was captured by the circTAF4B probe. **(D)** Schematic diagram of the dual-luciferase reporter plasmid targeting circTAF4B. **(E)** Luciferase activity was significantly decreased after cotransfection with circTAF4B wild-type (WT) and miR-1298-5p mimics. **(F, G)** qRT-PCR showed that knockdown of circTAF4B had no effect on the expression of miR-1298-5p, and miR-1298-5p overexpression had no effect on the expression of circTAF4B. Data represent the mean ± SD of more than three independent experiments. *p < 0.05; **p < 0.01.

To further investigate the mechanism of circTAF4B in BCa tumorigenesis, we carried out a nuclear/cytoplasmic fractionation assay and found that circTAF4B was mainly distributed in the cytoplasm of BC cells (**Figure 4A**), indicating that circTAF4B may act as a “miRNA sponge” to capture tumor suppressor miRNAs. To verify this hypothesis, we synthesized a circTAF4B-specific probe and found that it could indeed capture circTAF4B upon increased expression of circTAF4B (**Figure 4B**).

RNA pull-down assays showed that miR-1298-5p was significantly enriched in the circTAF4B probe in both BCa cell lines (**Figure 4C**). Furthermore, bioinformatics analysis predicted that circTAF4B was complementary to the seed sequence of miR-1298-5p. Then, luciferase reporter plasmids containing circTAF4B wild-type (WT) and mutant (Mut) were cotransfected with miR-1298-5p. A luciferase reporter assay showed that luciferase activity decreased significantly when the circTAF4B WT plasmid was cotransfected with miR-1298-5p (**Figures 4D, E**). Nevertheless, knockdown or overexpression of circTAF4B did not change the expression of miR-1298-5p (**Figures 4F, G**).

### circTAF4B Modulates TGFA by Sponging miR-1298-5p

**Figure 5:**
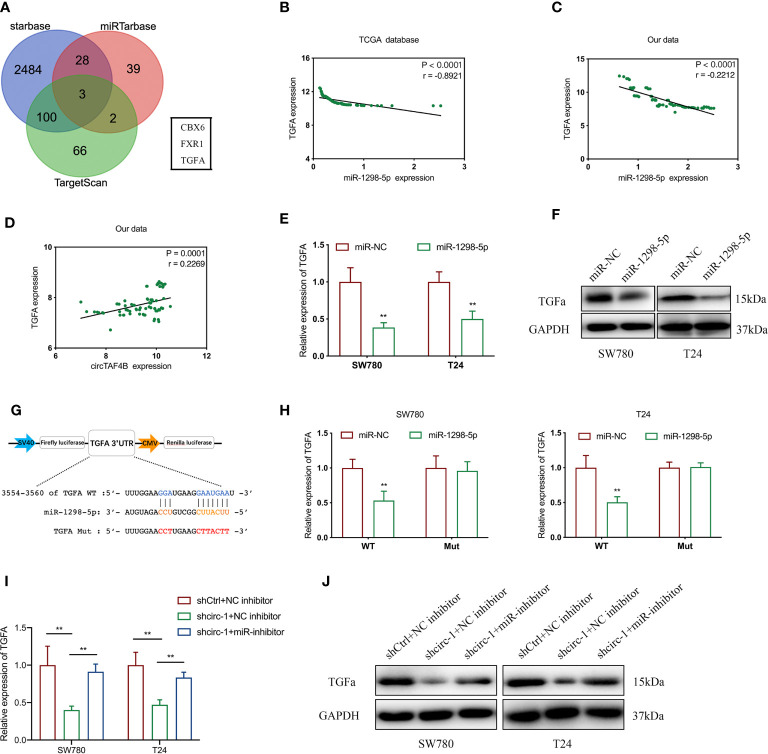
5circTAF4B positively regulates TGFA by sponging miR-1298-5p. **(A)** Venn diagram analysis of miR-1298-5p target genes by using three databases. **(B, C)** The TCGA database and our data showed that miR-1298-5p expression was negatively correlated with TGFA expression in BCa tissues. **(D)** Our data showed that circTAF4B expression was positively correlated with TGFA expression in Bca tissues. **(E, F)** Overexpression of miR-1298-5p decreased the expression of TGFA at both the mRNA and protein levels. **(G)** Schematic diagram of a dual-luciferase reporter plasmid constructed by TGFA sequence complementation with miR-1298-5p. **(H)** Dual-luciferase assay showed that miR-1298-5p reduced the luciferase activity of the vector containing wild-type TGFA. **(I, J)** Inhibition of miR-1298-5p reversed the mRNA and protein expression of TGFA suppression by circTAF4B knockdown. Data represent the mean ± SD of more than three independent experiments. *p < 0.05; **p < 0.01.

To investigate the target genes of miR-1298-5p, we used miRNA databases (miRtarbase, starBase and TargetScan) to forecast the potential target genes of miR-1298-5p and chose CBX6, FXR1 and TGFA for further experiments (**Figure 5A**). Using the TCGA database, we found that the expression level of miR-1298-5p (MIMAT0005800) in BCa tissues was lower than that in normal tissues (**Supplementary Figure S1A**), while the expression level of TGFA in BCa was significantly higher than that in normal tissues (**Supplementary Figure S1B**). miR-1298-5p expression was negatively correlated with TGFA expression in BCa tissues from the TCGA database (**Figure 5B**). The expression level of TGFA was negatively correlated with miR-1298-5p expression in 76 BCa tissues collected (**Figure 5C**). Moreover, circTAF4B expression was positively correlated with TGFA expression in 76 Bca tissues (**Figure 5D**). Further experiments confirmed that augmented expression of miR-1298-5p suppressed the mRNA and protein expression of TGFA (**Figures 5E, F**).

To further verify the core binding sites between miR-1298-5p and TGFA, we mutated the TGFA 3’UTR (3554-3560), which was predicted to be complementary to miR-1298-5p, and constructed a dual-luciferase reporter plasmid (**Figure 5G**).

Dual-luciferase reporter plasmids containing TGFA wild-type (WT) or mutant (Mut) were cotransfected with miR-1298-5p mimics. A dual-luciferase assay confirmed that miR-1298-5p remarkably reduced the luciferase activity of TGFA wild type (WT) (**Figure 5H**). Finally, qRT-PCR and western blotting experiments proved that inhibition of TGFA expression by circTAF4B knockdown was compensated for by inhibition of miR-1298-5p (**Figures 5I, J**). These results revealed that circTAF4B could upregulate TGFA expression by sponging miR-1298-5p.

### Overexpression of TGFA Reverses the Inhibition of BCa Cell Growth and Metastasis *In Vitro* Mediated by shcircTAF4B

**Figure 6:**
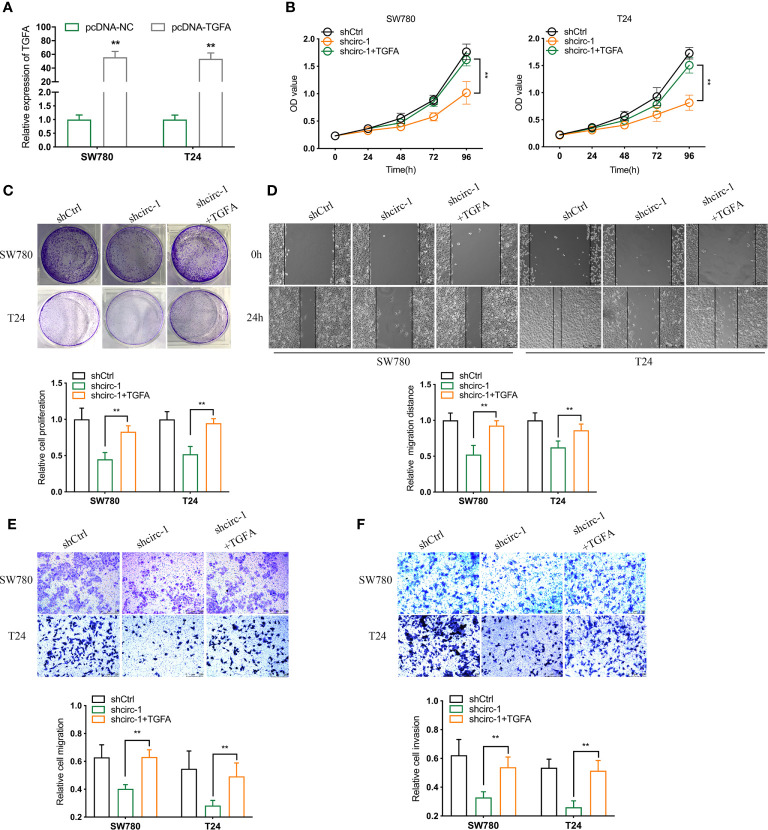
Overexpression of TGFA reverses the decline in BCa cell growth and metastasis *in vitro* induced by circTAF4B knockdown. **(A)** The effect of pcDNA3.1-TGFA was verified by qRT-PCR. **(B, C)** CCK-8 and colony formation assays showed that TGFA enhanced the reduction in cell proliferation caused by shcircTAF4B. **(D, E)** Wound healing and Transwell migration assays showed that TGFA reversed the cell migration reduction induced by shcircTAF4B. **(F)** Transwell invasion assays showed that TGFA reversed the inhibition of cell migration mediated by shcircTAF4B. Data represent the mean ± SD of more than three independent experiments. *p < 0.05; **p < 0.01.

To investigate whether circTAF4B promotes proliferation and metastasis by regulating TGFA expression, we conducted a rescue assay between circTAF4B and TGFA. TGFA expression in BCa cells was increased dramatically after transfection with pcDNA3.1-circTAF4B (**Figures 6A**, **B**). CCK-8 and colony formation experiments showed that forced expression of TGFA could obviously reverse the cell proliferation suppression induced by circTAF4B inhibition (**Figures 6B, C**). Wound healing and Transwell migration assays showed that knockdown of circTAF4B restrained BCa cell migration, while cotransfection of pcDNA3.1-TGFA impaired this effect (**Figures 6D, E**). Transwell invasion assays indicated that the cell invasion ability inhibited by shcircTAF4B was reversed significantly by enhancing TGFA expression (**Figure 6F**). The above results demonstrated that overexpression of TGFA reversed the suppression of BCa cell growth and metastasis induced by circTAF4B knockdown.

### Knockdown of circTAF4B Inhibited Tumor Growth and EMT of Bca *In Vivo*

**Figure 7:**
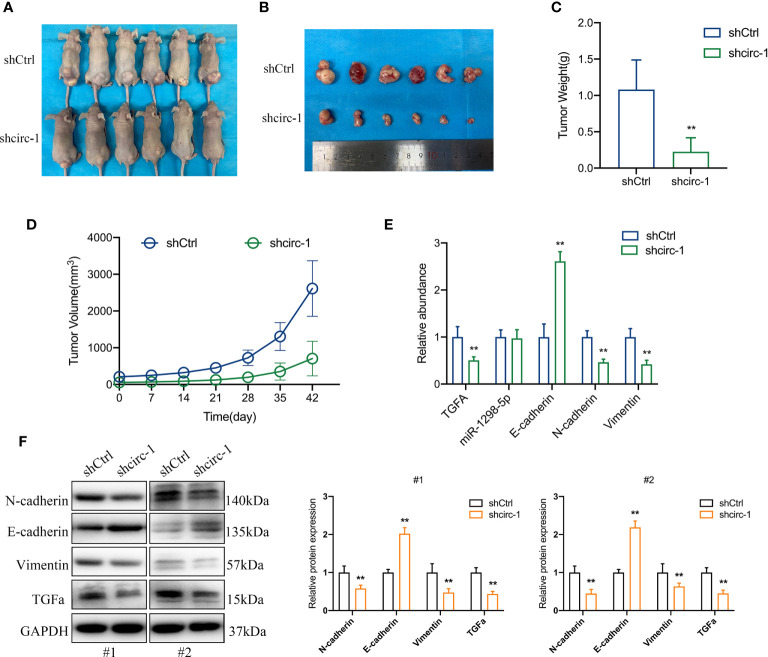
Silencing of circTAF4B suppressed the growth and EMT process of BCa cells *in vivo*. **(A)** Nude mice with transplanted tumors were sacrificed. **(B)** Transplanted tumors removed from two groups of nude mice. **(C)** The tumor weight of the circTAF4B-downregulated group was significantly lower than that of the negative control group. **(D)** The volume of tumors in both groups was measured weekly. **(E)** The expression levels of TGFA, miR-1298-5p and EMT markers in the circTAF4B downregulated group and negative control group. **(F)** western blotting assays showed that silencing circTAF4B downregulated N-cadherin, vimentin and TGFα expression and upregulated E-cadherin expression. Data represent the mean ± SD of more than three independent experiments. *p < 0.05; **p < 0.01.

**Figure 8:**
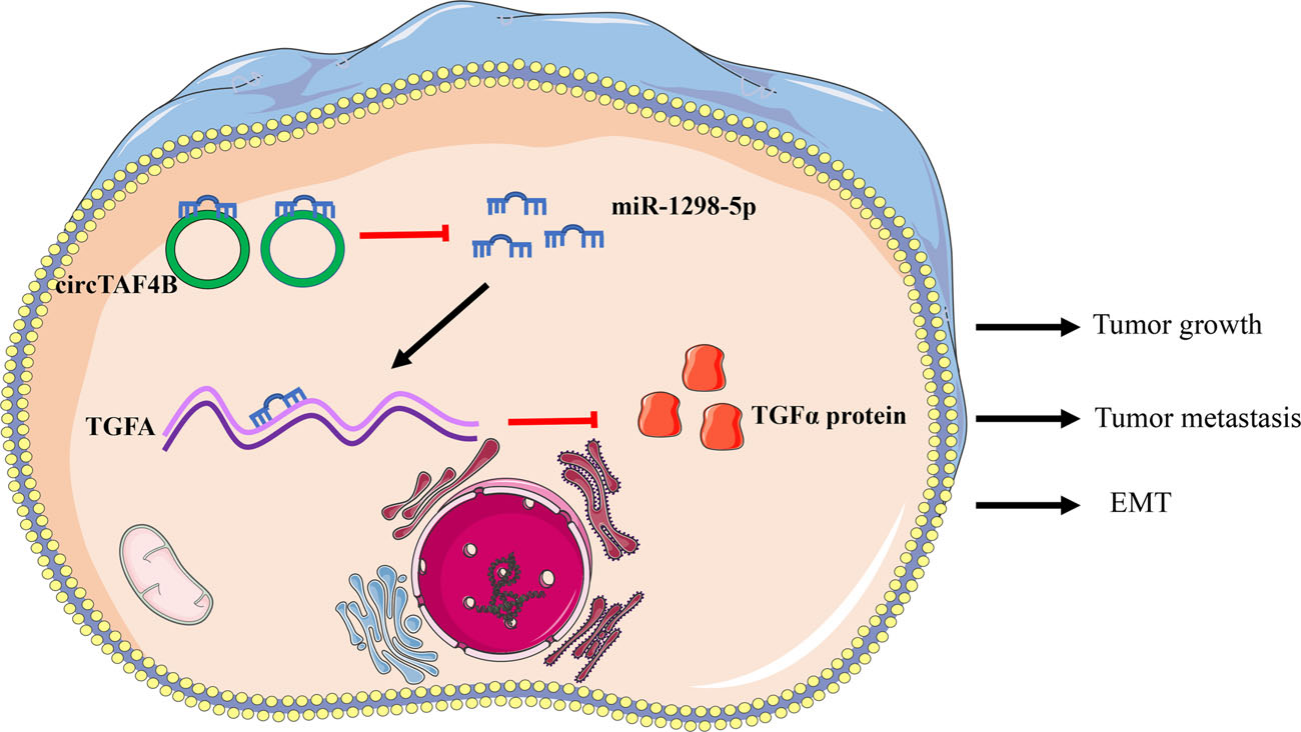
Schematic diagram of the circTAF4B/miR-1298-5p/TGFA axis in the progression of BCa.

To confirm the role of circTAF4B in Bca progression *in vivo*, we carried out tumor xenograft experiments. SW780 cells stably expressing shcricTAF4B were subcutaneously injected into the backs of nude mice. The nude mice were sacrificed, and the transplanted tumors were collected after 6 weeks (**Figures 7A, B**). As shown in **Figures 7C, D** the tumor volume and weight of tumors derived from the circTAF4B downregulated group were significantly lower than those in the negative control group. Furthermore, knockdown of circTAF4B inhibited the expression of TGFA, N-cadherin, and vimentin while upregulating the expression of E-cadherin *in vivo* (**Figure 7E**). Western blotting assays confirmed that downregulation of circTAF4B inhibited the protein expression levels of N-cadherin, vimentin and TGFα while increasing the protein expression level of E-cadherin (**Figure 7F**). In summary, circTAF4B positively regulates TGFA expression and promotes tumor growth and EMT *in vivo*. The mechanistic diagram of the circTAF4B/miR-1298-5p/TGFA axis is shown in **Figure 8**.

## Discussion

In recent years, with the development of RNA sequencing technology, a large number of circRNAs have been discovered (26, 27). Increasing evidence has shown that circRNAs play an important role in the occurrence and development of tumors by regulating the malignant phenotype of tumor cells (28–31). For example, circular RNA FAM114A2 suppresses the progression of bladder cancer by sponging miR-762 (32). The circular RNA circFNDC3B reduces bladder cancer progression through the miR-1178-3p/G3BP2/SRC/FAK axis (33). The circular RNA circ-DONSON enhances GC growth and invasion (34). In this study, we focused on a circRNA derived from 10-13 exons of TAF4B, which is named circTAF4B. A previous study suggested that circTAF4B was overexpressed in BCa tissues compared to matched normal tissues through a microarray dataset (GSE92675). Further analysis revealed that circTAF4B may interact with miR-515-5p to regulate the expression of several genes, including ﻿HIST1H3B, CENPA, ﻿HIST1H2B, ﻿HIST1H3H, ﻿HIST1H3F and ﻿HIST1H2BO (35). However, these results were based on bioinformatics analysis instead of rigorous experiments. Currently, we do not understand the role of circTAF4B in the progression of BCa. Hence, it is imperative to investigate the role of circTAF4B in the development of Bca.

Our study demonstrated that circTAF4B was significantly augmented in both BCa tissues and cell lines. Increased expression of circTAF4B was positively correlated with the pathological grade of BCa and negatively correlated with the survival rate of BCa patients. Furthermore, we found that circTAF4B accelerated the growth, metastasis and EMT of BCa cells *in vitro* and *in vivo*.

A previous study suggested that circRNAs originating from exons are mostly distributed in the cytoplasm. They may exert their roles by acting as “miRNA sponges” or translating proteins (36). In this study, we discovered that circTAF4B was mainly distributed in the cytoplasm *via* a nuclear/cytoplasmic fractionation assay. However, circTAF4B lacks an open reading flame (ORF) and protein translation ability based on the analysis of the cirRNADB database. Hence, circTAF4B may act as a “miRNA sponge” during the progression of Bca. RNA pull-down and dual-luciferase assays demonstrated that circTAF4B positively sponged miR-1298-5p. Nevertheless, knockdown of circTAF4B could not modulate the expression of miR-129-5p, indicating that circTAF4B only absorbs miR-129-5p and inhibits its activity instead of facilitating its degradation.

MiRNAs are a type of long noncoding RNA 19-24 nt in length. They can bind to the 3’UTRs of their target genes to prevent the transcription and translation process. MiRNAs have been demonstrated to play a crucial role in the growth and metastasis of various tumors (20, 37).

It has been reported that miRNA-1298-5p inhibits the progression of bladder cancer by abolishing the expression of connexin 43 (22). In our study, we speculated that TGFA might be the target gene of miR-1298-5p during BCa progression through bioinformatics analysis. Further experiments suggested that miR-1298-5p bound to the 3’UTR of TGFA and inhibited the mRNA and protein expression of TGFA.

TGFA, a member of the epidermal growth factor family, is encoded by the TGFA gene. TGFA can activate a series of signaling pathways by binding with EGFR to regulate cellular biological processes such as cell proliferation, metastasis, differentiation, tissue repair, wound repair and energy metabolism (38). TGFA has been reported to accelerate cell proliferation, invasion and EMT in breast cancer, prostate cancer and hepatic cancer (39–41) and modulate lung cancer EMT through regulation by PHD3 (42). In addition, miR-152 can inhibit migration and invasion by targeting TGFA in prostate cancer (43). miR-376c can reduce the progression of osteosarcoma by targeting TGFA (44). miR-505 suppressed tumorigenesis of endometrial cancer by targeting TGFA (45). These studies suggest that TGFA is frequently upregulated in malignant tumors and modulated by miRNAs. In our study, we discovered that circTAF4B promoted the growth and metastasis of BCa by increasing TGFA expression. Induced expression of TGFA reversed cell growth and suppression of metastasis caused by circTAF4B downregulation. Based on the above results, we demonstrated that circTAF4 acted as the “miRNA sponge” for miR-1298-5p to increase TGFA expression.

In conclusion, we identified a novel circular RNA, termed circTAF4B, that acted as an oncogene in Bca. circTAF4B could promote cell proliferation, migration, invasion and EMT in BCa by modulating the miR-1298-5p/TGFA axis. Our study may open up a new area of research in circRNA studies and provide a novel target for the diagnosis and treatment of BCa.

## Data Availability Statement

The original contributions presented in the study are included in the article/**Supplementary Material**. Further inquiries can be directed to the corresponding authors.

## Ethics Statement

The studies involving human participants were reviewed and approved by the Regional Committee for Medical Research Ethics and the Human Ethics Committee of Peking University Shenzhen Hospital. The patients/participants provided their written informed consent to participate in this study. The animal study was reviewed and approved by Ethics Committee of Peking University Shenzhen Hospital.

## Author Contributions

CCH, XFL and XF designed the research and performed the experiments. MLY analyzed the data. GCQ collected the Bca tissues. CCH wrote the draft and modified the manuscript. GHC and XTZ supervised the study and provided the funding. All authors contributed to the article and approved the submitted version.

## Funding

This study was supported by National Natural Science Foundation of China (81801517 to XFL), Shenzhen Project of Science and Technology (Grant No. JCYJ20190809094407602) to XFL, Shenzhen Project of Science and Technology (Grant No. JCYJ20180302145109198) to XTZ, Medjaden Academy & Research Foundation for Young Scientists (Grant No. MJR20190009) to XFL and the fund of “San-ming” Project of Medicine in Shenzhen (NO.SZSM201812088).

## Conflict of Interest

The authors declare that the research was conducted in the absence of any commercial or financial relationships that could be construed as a potential conflict of interest.
