# Supplementary material for: Circular RNA TAF4B Promotes Bladder Cancer Progression by Sponging miR-1298-5p and Regulating TGFA Expression
Source: Front Oncol. 2021 Jul 12;11:643362. doi: 10.3389/fonc.2021.643362 (PMC8312550; doi:10.3389/fonc.2021.643362)
Supplement: Supplementary file 1 [file DataSheet_1.docx]

**Supplementary Table S1.** List of primers for qRT-PCR

| Gene | Sequence (5’-3’) | |
| --- | --- | --- |
| circTAF4B | F: taaggcagccaagagtcgtt | R: taaggttgacccctgccata |
| TAF4B | F: gccagtcctatcgagacgtt | R: tgacaagcatgtcccctcac |
| GAPDH | F: aatgggcagccgttaggaaa | R: gcgcccaatacgaccaaatc |
| U6 | F: gcttcggcagcacatatactaaaat | R: cgcttcacgaatttgcgtgtcat |
| miR-1227-5p | F: tggggccaggcg | R: gtccagtttttttttttttttccac |
| miR-1265 | F: gcaggatgtggtcaagtg | R: ggtccagtttttttttttttttaacaac |
| miR-1298-5p | F: gttcattcggctgtccag | R: ggtccagtttttttttttttttacatc |
| miR-1299 | F: cagttctggaattctgtgtga | R: gtccagtttttttttttttttccct |
| miR-1305 | F: gcagttttcaactctaatggga | R: ggtccagtttttttttttttttctct |
| miR-223-5p | F: gcagcgtgtatttgacaag | R: gtccagtttttttttttttttaactcag |
| miR-224-5p | F: cagtcaagtcactagtggttc | R: gtccagtttttttttttttttctaaacg |
| miR-498-5p | F: gccagggggcgt | R: aggtccagtttttttttttttttgaa |
| miR-513a-5p | F: cagttcacagggaggtg | R: ggtccagtttttttttttttttatgac |
| miR-515-5p | F: cagttctccaaaagaaagcac | R: gtccagtttttttttttttttcagaaag |

**Supplementary Table S2.** List of sequences for shRNAs

| shRNA | Sequence (5’ -3’) |
| --- | --- |
| circTAF4B shRNA-1(shcirc-1) | gaaagccaaagagagatgagg |
| circTAF4B shRNA-2(shcirc-2) | gccaaagagagatgaggatga |

**Supplementary Table S3**. Clinical data of 76 patients with bladder cancer.

| Number | Age | sex | grade | T | N | Number | Age | sex | grade | T | N |
| --- | --- | --- | --- | --- | --- | --- | --- | --- | --- | --- | --- |
| 1 | 59 | male | low | T3 | N1 | 39 | 38 | male | low | T4 | N0 |
| 2 | 70 | male | low | T2 | N0 | 40 | 59 | male | low | T3 | N0 |
| 3 | 54 | male | low | T3 | N0 | 41 | 58 | male | low | T2 | N0 |
| 4 | 55 | male | low | T2 | N0 | 42 | 48 | female | low | T2 | N0 |
| 5 | 76 | female | low | T2 | N0 | 43 | 67 | female | low | T2 | N0 |
| 6 | 72 | female | low | T3 | N0 | 44 | 49 | female | low | T2 | N0 |
| 7 | 79 | female | high | T1 | N0 | 45 | 57 | male | low | T3 | N0 |
| 8 | 51 | female | high | T2 | N0 | 46 | 57 | male | low | T3 | N0 |
| 9 | 71 | male | high | T2 | N0 | 47 | 55 | male | low | T3 | N0 |
| 10 | 40 | male | high | T2 | N0 | 48 | 56 | male | low | T3 | N0 |
| 11 | 56 | female | high | T1 | N0 | 49 | 47 | male | low | T3 | N0 |
| 12 | 56 | male | high | T3 | N0 | 50 | 49 | male | low | T2 | N0 |
| 13 | 53 | male | high | T3 | N0 | 51 | 45 | male | high | T2 | N0 |
| 14 | 50 | female | high | T3 | N0 | 52 | 59 | male | high | T2 | N0 |
| 15 | 50 | female | high | T4 | N0 | 53 | 58 | male | high | T2 | N0 |
| 16 | 57 | female | low | T4 | N0 | 54 | 56 | male | high | T2 | N0 |
| 17 | 59 | male | low | T3 | N0 | 55 | 74 | male | high | T2 | N0 |
| 18 | 55 | male | low | T2 | N0 | 56 | 79 | male | high | T2 | N0 |
| 19 | 62 | female | high | T3 | N0 | 57 | 81 | female | high | T2 | N0 |
| 20 | 60 | male | high | T3 | N0 | 58 | 50 | male | high | T2 | N0 |
| 21 | 74 | male | high | T3 | N0 | 59 | 52 | male | high | T2 | N0 |
| 22 | 68 | male | high | T3 | N0 | 60 | 53 | male | high | T1 | N0 |
| 23 | 69 | male | high | T3 | N0 | 61 | 80 | female | high | T2 | N0 |
| 24 | 68 | male | high | T2 | N0 | 62 | 65 | male | high | T2 | N0 |
| 25 | 64 | female | high | T3 | N0 | 63 | 49 | female | high | T2 | N0 |
| 26 | 65 | male | high | T1 | N0 | 64 | 75 | female | high | T2 | N0 |
| 27 | 50 | female | high | T3 | N0 | 65 | 76 | male | high | T2 | N0 |
| 28 | 53 | male | high | T3 | N0 | 66 | 58 | female | high | T2 | N0 |
| 29 | 53 | male | high | T3 | N0 | 67 | 60 | female | high | T2 | N0 |
| 30 | 69 | male | low | T2 | N0 | 68 | 67 | male | high | T2 | N0 |
| 31 | 72 | female | high | T3 | N0 | 69 | 68 | female | high | T2 | N0 |
| 32 | 77 | male | high | T3 | N0 | 70 | 69 | female | high | T2 | N0 |
| 33 | 69 | female | low | T2 | N0 | 71 | 70 | male | high | T2 | N0 |
| 34 | 67 | male | high | T3 | N0 | 72 | 70 | male | high | T2 | N0 |
| 35 | 71 | male | low | T4 | N0 | 73 | 60 | male | low | T2 | N0 |
| 36 | 80 | female | low | T2 | N0 | 74 | 72 | male | high | T2 | N0 |
| 37 | 72 | female | high | T3 | N0 | 75 | 73 | male | high | T2 | N0 |
| 38 | 75 | female | high | T1 | N0 | 76 | 76 | female | low | T2 | N0 |

**Supplementary Figure S1.** Original data from TCGA database.





**Supplementary Figure S1.** Original data regarding miR-1298-5p and TGFA in BC from TCGA database. A, In TCGA database, the expression level of miR-1298-5p (MIMAT0005800) in bladder cancer samples was significantly lower than that in normal tissues. B, The expression of TGFA in bladder cancer samples in TCGA database was significantly higher than that in normal tissues.
